# Bioprospecting Deep-Sea Actinobacteria for Novel Anti-infective Natural Products

**DOI:** 10.3389/fmicb.2018.00787

**Published:** 2018-04-30

**Authors:** Dongbo Xu, Linna Han, Chunhui Li, Qi Cao, Duolong Zhu, Nolan H. Barrett, Dedra Harmody, Jing Chen, Haining Zhu, Peter J. McCarthy, Xingmin Sun, Guojun Wang

**Affiliations:** ^1^Harbor Branch Oceanographic Institute, Florida Atlantic University, Fort Pierce, FL, United States; ^2^Department of Molecular Medicine, Morsani College of Medicine, University of South Florida, Tampa, FL, United States; ^3^Infection Control Center, Xiangya Hospital, Central South University, Changsha, China; ^4^Department of Molecular and Cellular Biochemistry, University of Kentucky, Lexington, KY, United States

**Keywords:** actinobacteria, natural products, anti-infective, antifungal, antibacterial, drug resistance, lanthanum chloride, deep-sea sponge

## Abstract

The global prevalence of drug resistance has created an urgent need for the discovery of novel anti-infective drugs. The major source of antibiotics in current clinical practice is terrestrial actinobacteria; the less-exploited deep-sea actinobacteria may serve as an unprecedented source of novel natural products. In this study, we evaluated 50 actinobacteria strains derived from diverse deep water sponges and environmental niches for their anti-microbial activities against a panel of pathogens including *Candida albicans*, *Clostridium difficile*, *Staphylococcus aureus*, and methicillin-resistant *S. aureus* (MRSA), and *Pseudomonas aeruginosa*. More than half of the tested strains (27) were identified as active in at least one assay. The rare earth salt lanthanum chloride (LaCl_3_) was shown to be as an effective elicitor. Among the 27 strains, the anti-microbial activity of 15 were induced or enhanced by the addition of LaCl_3_. This part of study focused on one strain R818, in which potent antifungal activity was induced by the addition of LaCl_3_. We found that the LaCl_3_-activated metabolites in R818 are likely antimycin-type compounds. One of them, compound **1**, has been purified. Spectroscopic analyses including HR-MS and 1D NMR indicated that this compound is urauchimycin D. The antifungal activity of compound **1** was confirmed with a minimal inhibitory concentration (MIC) of 25 μg/mL; the purified compound also showed a moderate activity against *C. difficile*. Additional notable strains are: strain N217 which showed both antifungal and antibacterial (including *P. aeruginosa*) activities and strain M864 which showed potent activity against *C. difficile* with an MIC value (0.125 μg/mL) lower than those of vancomycin and metronidazole. Our preliminary studies show that deep-sea actinobacteria is a promising source of anti-infective natural products.

## Introduction

Infectious diseases remain a major threat to human health, annually causing millions of deaths worldwide, especially in medically less-developed countries and regions (Spellberg et al., 2008). There were estimated 1.2 million tuberculosis deaths, 1.03 million HIV/AIDS deaths, and 719,600 malaria deaths in 2016 (The Lancet, 2017). However, the situation is worsened significantly by the prevalence of (multi-)drug resistance (Ventola, 2015). Due to drug resistance, current antibiotics are losing their capacity to treat infectious diseases, and pathogens such as *Klebsiella pneumoniae*, *Pseudomonas aeruginosa*, and *Staphylococcus aureus* have again become fatal threats. Both the United States Centers for Disease Control and Prevention (CDC) and the World Health Organization (WHO) have issued lists of priority pathogens: the CDC listed 18 drug-resistant bacteria as threats to the United States^1^; in 2017, WHO listed, for the first time, 12 families of bacteria as the greatest threat to human health^2^. Novel antibiotics are urgently needed to treat disease caused by these pathogens.

Actinobacteria, a group of Gram-positive filamentous bacteria, have been an exceptionally rich source of bioactive natural products used to treat infectious diseases and are the source of the majority of currently used antibiotics (Newman and Cragg, 2016). The genomic era has witnessed an explosion of genomic data that unexpectedly revealed the abundance of silent/cryptic secondary metabolic gene clusters in actinomycete genomes, which are unexpressed under standard laboratory culture conditions. Activation of these silent pathways represents a tremendous opportunity to discover new compounds to treat infectious and other diseases from known organisms (Van Lanen and Shen, 2006; Nett et al., 2009; Bachmann et al., 2014; Ziemert et al., 2016). Meanwhile, actinobacteria from unique environmental niches or new taxa are still highly sought for drug discovery. Due to sampling difficulties, deep-sea actinobacteria are generally much less studied as a source of natural products. In this study, we focused on actinobacteria isolated from deep-sea sponges and aimed to find novel anti-microbial natural products. Actinobacteria were cultured and tested against a panel of bacterial pathogens regarded as common causes of healthcare-associated infections and listed among the most severe threats to human health by CDC^1^ or WHO^2^: *Clostridium difficile*, *P. aeruginosa*, methicillin-resistant *S. aureus* (MRSA), and *Candida albicans*. *C. difficile*, a cause of life-threatening diarrhea, is listed by CDC as an URGENT threat (the highest level). The *C. difficile* infection (CDI) is the most common cause of infectious diarrhea in the healthcare setting with about 453,000 cases and 29,000 deaths yearly in the United States as reported by CDC in 2015; the annual healthcare costs for acute care facilities alone are about $4.8 billion^3^. Though therapeutics such as vancomycin, metronidazole, fidaxomicin, or nitazoxanide are available, due to drug resistance or toxicity to gut microbiome, new drugs, especially those with narrow spectrum, are highly needed. As a CRITICAL pathogen (the highest level) listed by WHO, *P. aeruginosa* is a leading cause of hospital-associated infections (HAIs); many isolates are resistant to a wide range of antibiotics; multidrug-resistant *P. aeruginosa* is also listed as a SERIOUS threat (the second highest level) by CDC. Also listed as SERIOUS threats by CDC include both fluconazole-resistant *Candida* and MRSA, the latter is regarded as a HIGH (the second highest level) by WHO as well.

In this preliminary study, we evaluated anti-pathogen activities of crude extracts from 50 deep-sea actinobacteria strains derived from various sponge hosts that were collected from environmentally and geographically diverse locations; many of them are rare actinobacteria. In an attempt to activate any dormant secondary metabolic capabilities of these strains, LaCl_3_ was supplemented to the culture medium as an elicitor. LaCl_3_ has been shown to be an effective elicitor of secondary metabolism in microorganisms in our previous studies (Kawai et al., 2007; Tanaka et al., 2010; Ochi and Hosaka, 2013; Ochi et al., 2014). The efficient activation/induction of new metabolites/anti-microbial activities by LaCl_3_ was detected in this study. Strains exhibiting potent antifungal or antibacterial activities were identified; of particular interest is the identification of a strain producing metabolites which are more potent than vancomycin against *C. difficile*.

## Materials and Methods

### Strains, Media, and Chemicals

All marine actinobacteria are maintained in the Harbor Branch Oceanographic Institute (HBOI) Marine Microbial Culture Collection. GYM, SFM, and SPY (also called Medium A) media were prepared as previously described (Hu and Ochi, 2001; Wang et al., 2008; Tanaka et al., 2009). All actinobacteria strains were cultured at 25°C. Test pathogens included *C. albicans* ATCC 44506, *S. aureus* ATCC 29213 and MRSA ATCC 700787, *P. aeruginosa* ATCC 27853, and *C. difficile* UK6.

Chemicals and organic solvents were purchased from Thermo-Fisher Scientific or Sigma-Aldrich. Premixed LB powder was purchased from BD Difco. Nystatin (100 U) and gentamicin (10 μg) disks were manufactured by Becton-Dickinson BBL; cefoxitin (30 μg) disks were manufactured by Oxoid.

### Fermentation, Extraction, and HPLC Analysis of Actinobacteria Metabolites

A small-scale 100 mL fermentation was used. Each strain was inoculated into two 250-mL flasks, each containing 100 mL of SPY medium with or without the supplementation of 2 mM LaCl_3_, and cultured at 25°C on a rotary shaker (220 rpm) for 7 days. Each fermentation broth was mixed with 200 mL ethyl acetate (EtOAc), and subjected to 60 min ultrasonication, with mixing every 20 min; the extraction was repeated once using fresh EtOAc. The organic layers were combined and evaporated under vacuum using a Heidolph evaporator to generate a crude extract. Dried crude extracts were stored at -20°C for HPLC analysis and bioassays.

Each crude extract was dissolved in methanol (100%) to a final concentration of 5 or 10 mg/mL. HPLC analysis was performed using an UltiMate 3000 system (Thermo) equipped with an Apollo C18 column (250 mm × 4.5 mm) with a fingerprint gradient of H_2_O+0.1% TFA (solvent A)/MeCN (solvent B): 5 min equilibration, 5% B; 0 min, 5% B; 15 min, 100% B; 20 min, 100% B, and a flow rate of 1.5 mL/min.

### Extraction and Purification of LaCl_3_-Activated Metabolites With Antifungal Activity in the Strain R818

R818 spores from Marine Agar 2216 (MA) plates were inoculated into 60 250-mL flasks; each flask contains 100 mL of SPY media (soluble starch 20 g, glucose 10 g, peptone 5 g, yeast extract 5 g, K_2_HPO_4_ 0.5 g, MgSO_4_⋅7H_2_O 0.5 g, CaCO_3_ 2 g, and sea salt 39.5 g, per liter) supplemented with 2 mM LaCl_3_. Flasks were incubated on a shaker at 200 rpm and 28°C for 7 days. The broth (6 L) was extracted with four times with an equal volume of EtOAc, and the combined EtOAc layers were concentrated under vacuum. The crude extract (4.9 g) was fractioned on a CombiFlash Rf200 system (Teledyne Isco) using a RediSep Rf Gold C18 column (size 50 g) with a flow rate of 40 mL/min. Fractions with antifungal activity were pooled and used for bioactivity-guided purification. Compound **1** (2.0 mg) was purified by semi-preparative HPLC (H_2_O+0.1% TFA/MeCN: 70/30, flow rate at 3 mL/min) using an Apollo C18 column (250 mm × 10 mm).

### Structural Elucidation of Compound **1**

The structure of compound **1** was determined by HRESI-MS and 1D NMR spectra. The exact molecular weight was determined by an LTQ Orbitrap VELOS high-resolution mass spectrometer. 1D NMR spectra were recorded on a JEOL ECA-600 system using a Shigemi symmetrical NMR microtube with the solvent DMSO-*d6*.

### Anti-microbial Bioassays

Activity against *C. albicans*, *S. aureus*, MRSA, and *P. aeruginosa* assays was determined using a standard disk-diffusion method as described previously (Wright et al., 2007). Briefly, 125 μg of each sample was applied to a 6-mm diameter filter-paper disk, which was then dried and placed onto the surface of a seeded agar plate: Sabouraud Dextrose agar plates for *C. albicans* and cation-supplemented Mueller–Hinton agar plates for bacteria. All plates were seeded at approximately 1 × 10^6^ cells/mL. Zones of growth inhibition were measured after incubation for 24 h at 37°C. Positive controls were included for all assays: nystatin (100 U) for *C. albicans*; cefoxitin (30 μg) for MRSA; and gentamicin (10 μg) for *S. aureus* and *P. aeruginosa*.

Activity against *C. difficile* was determined using an initial screening against UK6, a hypervirulent epidemic strain (Wang et al., 2015). Extracts that showed sensitivity against UK6 at 64 μg/mL were further proceeded to determine minimal inhibitory concentrations (MICs) using the broth microdilution method (Clinical and Laboratory Standards Institute, 2007). Briefly, actinobacteria extracts were added at final concentrations ranging from 64 to 0.0625 μg/mL to wells of 96-well microplates which contain UK6 cells (1.5 × 10^8^ cells/mL, 100 μL per well) in the BHIS medium. The plates were incubated at 37°C for 24 h. The MIC value of each extract was determined as the lowest concentration at which no growth of UK6 was observed. Vancomycin and metronidazole were included as positive controls.

### MTT Cell Viability Assay

MTT (3-(4,5-dimethylthiazol-2yl)-2,5-dipheynyltetrazolium bromide (Sigma-Aldrich, St. Louis, MO, United States) cell viability assay was performed to evaluate the cytotoxicity of extracts against HepG2 and HEK293T cell lines cultured in Dulbecco’s Modified Eagle Medium (DMEM) supplied with 10% FBS and 1% penicillin/streptomycin. Cells (10^4^ cells/ well) were seeded in triplicates in 96-well plates and were cultured overnight. Extracts of the M864 strain were added to the 96-well plates at a final concentration ranging from 128 to 0.125 μg/mL in DMEM medium. Methanol solvent at a final concentration of 0.5% was used as a control. After 24-h incubation, MTT analysis of the plates was performed as described early (Fotakis and Timbrell, 2006). Data were analyzed with GraphPad PRISM 6 software (GraphPad Software, Inc., La Jolla, CA, United States), and the half maximal inhibitory concentration (IC_50_) was reported as the concentration of extract required for 50% inhibition compared with control cells.

## Results and Discussion

### Actinobacteria Isolated From Deep-Sea Sponges

With over 1,000 new compounds discovered annually over the last decade, marine natural products (MNPs) represent an increasingly attractive source of new anti-infective agents (Blunt et al., 2008, 2009, 2010, 2011, 2012, 2013, 2014, 2015, 2016, 2017). Among marine organisms, marine actinobacteria are an important producer (Manivasagan et al., 2014; Betancur et al., 2017). Over the past 30 years, HBOI has collected biological (such as sponges and corals) or sediment samples mainly from the east coast of the United States, the Gulf of Mexico, and the Caribbean Sea, as well as European and African deep waters using the Johnson Sea Link manned submersibles (Sfanos et al., 2005; Gaskill, 2011). From these samples, we have been isolating microorganisms including actinobacteria. In an effort to prospect these marine actinobacteria for novel anti-infective natural products and to optimize the approach for compound production, 50 strains from the HBOI collection were used in this study (see details in Supplementary Table S1), all of which were cultivated from marine sponges. With the exception of *Ircinia felix*, all sponge samples were collected from deep-sea environments ranging from ∼200 to ∼2,800 fsw, as summarized in **Table 1**. Analysis of strain taxonomy showed that the 50 strains represent 15 genera. Even though 19 strains are *Streptomyces* spp., most of the remaining strains are rare actinobacteria, such as *Actinomycetospora*, *Agrococcus*, *Leifsonia*, *Nocardiopsis*, *Promicromonospora*, *Rhodococcus*, *Salinispora*, and *Tsukamurella* (Supplementary Table S1).

**Table 1:** A summary of sponge species, the depth of sampling site, and the number of microbial strains isolated from each sponge.

Sponge taxonomy	Depth (fsw)	Sample location	Number of strains
*Axinellida* sp.	246	Gulf of Mexico, Florida, United States	1 (1)^∗^
*Sarcotagus* sp. or *Smenospongia* sp.^∗∗^	205	Georgia, United States	1
*Discodermia* sp.	440–575	Bahamas; Honduras; Guanaja	12 (6)
*Forcepia* sp.	230–240	Gulf of Mexico, Florida, United States	18 (14)
*Gorgonacea* sp.	1,123	Curacao	1
*Hexactinellida* + *Zoanthidea*	720	Curacao	2
*Ircinia felix*	20	Florida Keys, United States	1 (1)
*Leiodermatium* sp.	1,288	Puerto Rico; Florida (Miami), United States	5
*Oceanapiidae* sp.	2,790	Bahamas	1 (1)
*Scleritoderma cyanea*	795	Curacao	1 (1)
*Spongosorites* sp.	730	Puerto Rico	2 (1)
*Theonella* sp.	692	Puerto Rico	3 (1)
*Theonellidae* n.sp.	655	Florida Keys, United States	1 (1)
*Thrinacophora funiformis*	150	Florida (Key Biscayne), United States	1

*^∗^(x) indicates the number of strains that showed anti-infective activity in at least one assay. Several sponges have been collected from multiple locations. ^∗∗^The taxonomy of this sponge specimen (log# 23-V-92-1-011) was unable to be distinguished between these two species. fsw, feet sea water.*

### Deep-Sea Actinobacteria as a Rich Source of Anti-infective Natural Products

**Table 1** shows the diversity of both sponge species and environment where the sponge samples were obtained. This diverse source of actinobacteria might suggest a high degree of chemical diversity of secondary metabolites generated by these strains, in particular, among the less-exploited rare actinobacteria.

Since recent advances in the study of microbial genomes have shown an abundance of cryptic secondary metabolic gene clusters in the microbial genome, activation of these gene clusters might lead to discovery of new natural products. We have reported that salts of the rare earth element lanthanum have been reported to elicit the production of natural products (Kawai et al., 2007); the method was also used by other groups to identify new compounds, such as frenolicin G from *Streptomyces* sp. RM-4-15 which was isolated from an Appalachian active coal fire site (Wang et al., 2013). For each strain, LaCl_3_ (2 mM) was supplemented to the medium as a chemical elicitor. Fermentation broths were extracted with ethyl acetate, dried, and re-suspended in methanol for evaluation. As a result, a total of 100 extracts were tested for their activity against a panel of human pathogens.

**FIGURE 1:**
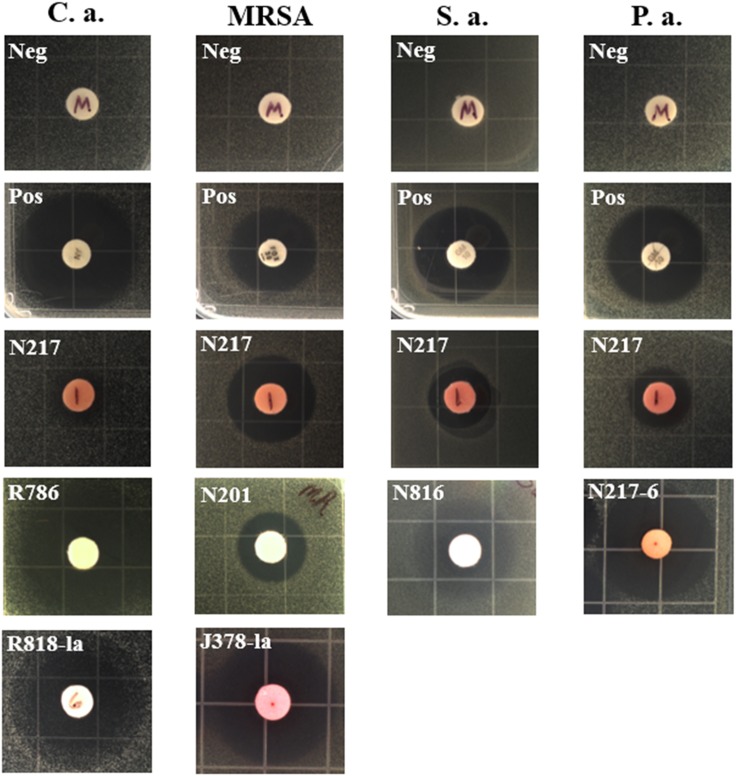
Representative inhibition zones in disk fusion assays. C.a., *Candida albicans*; S.a., *Staphylococcus aureus*; P.a., *Pseudomonas aeruginosa*, Neg, solvent methanol as negative controls; Pos, positive controls; nystatin (100 U) for C. *albicans*, gentamicin (10 μg) for *S. aureus* and *P. aeruginosa*, and cefoxitin (30 μg) for MRSA. N217, R786, N201, and N816, crude extract of each strain in the absence of LaCl_3_; R818-la and J378-la, crude extract of R818 and J378 supplemented with LaCl_3_ (2 mM), respectively; N217-6, a partially purified fraction (#6) of the N217 crude extract.

Due to the difficulty of sampling, deep-sea actinobacteria have been studied to a far lesser extent than those from shallow water and terrestrial sources, and thus one might expect them to have potential for the production of novel natural products. Our results indeed indicated a high rate of pathogen inhibiting activity. Out of 50 strains tested, 27 were identified as active in at least one assay. Of these, most strains (21) showed activity against the drug-resistant bacterium MRSA with a few strains showing potent anti-MRSA activity compared to the positive control (30 μg of cefoxitin and 19 mm of inhibition zone); metabolites of 11 strains showed antifungal activity; three strains exhibited anti-*Pseudomonas* activity and one strain showed potent activity against *C. difficile* (see full results in Supplementary Table S1 and representative bioassay results in **Figure 1** and **Table 2**).

**Table 2:** Representative strains in the disk diffusion assay against *Candida albicans*, *Staphylococcus aureus* and MRSA, and *Pseudomonas aeruginosa*. Inhibition is recorded as the diameter of the zone of growth inhibition (mm).

Strain	Inhibition zone (mm)			
	C.a.	MRSA	S.a.	P.a.
J378	ND	12	9	ND
J378-la	ND	25	16	ND
N201	10^1^	16	-^2^	9
N201-la	ND	13	-^2^	ND
N203	12	18	-^2^	14
N203-la	ND	11	-^2^	ND
N217	11	20	14	14
N217-la	ND	16	13	10
N248	ND	ND	-^2^	ND
N248-la	ND	10	-^2^	ND
N816	ND	ND	ND	ND
N816-la	ND	11	12	ND^3^
P114	ND	9	ND	ND
P114-la	13^1^	10	ND	ND
P257	ND	ND	12	ND
P257-la	ND	ND	26	ND
R786	22	12^1^	ND	ND
R786-la	22	13^1^	ND	ND
R818	ND	ND	-^2^	ND
R818-la	21	ND	-^2^	ND
S355	ND	ND	-^2^	ND
S355-la	ND	10^1^	-^2^	ND
V324	ND	11	-^2^	ND
V324-la	ND	15	-^2^	ND
Pos. cont.	27	19	22	23

*C.a., C. albicans; S.a., S. aureus; P.a., P. aeruginosa; Pos. cont., positive controls [nystatin (100 U) for C. albicans; cefoxitin (30 μg) for MRSA; gentamicin (10 μg) for S. aureus and P. aeruginosa]; “-la,” cultures supplemented with LaCl_3_; ND, activity not detected; ^1^the edge of inhibition zone is hazy; ^2^not determined; ^3^anti-P.a. activity was observed only using partially purified fractions.*

Two strains, R786 and R818, showed strong antifungal activity (**Table 2**). Notably, strain N217 has broad activity against all tested pathogens except *C. difficile*. Recent fractionation studies of N217 metabolites suggest that this broad spectrum activity is likely contributed by different types of compounds (Xu and Wang, unpublished data). The other notable strain is M864, which is the only strain producing potent anti-*C. difficile* metabolites in this study. Significantly, the MIC value (0.125 μg/mL) of extracts from the strain M864 is lower than those of vancomycin and metronidazole, both of which are first-line antibiotics used in the treatment of CDI (see details below). M864 was cultivated from a sponge of the family *Oceanapiidae* collected from Bahamas at a depth of 2790 ft. Sequence analysis of the M864 16S rDNA gene using BLAST showed 99% homology to that of *Salinispora arenicola* strain SCSIOZ-SH11 (GenBank acc# KC747479.1).

### Induction of Antifungal and Antibacterial Activity by LaCl_3_ Supplementation

LaCl_3_ has been shown to be an effective elicitor of secondary metabolism in microorganisms. Among 27 strains active in at least one assay, the addition of LaCl_3_ (2 mM) induced or enhanced the production of anti-microbial activity in 15 strains; in 11 strains, the activity was attenuated (see the details in Supplementary Table S2). For example, the strain R818 showed potent antifungal activity only in the presence of LaCl_3_; anti-MRSA activity was elicited in strains N816 and S355 with the addition of LaCl_3_, and was significantly enhanced in J378 (**Table 2**).

**FIGURE 2:**
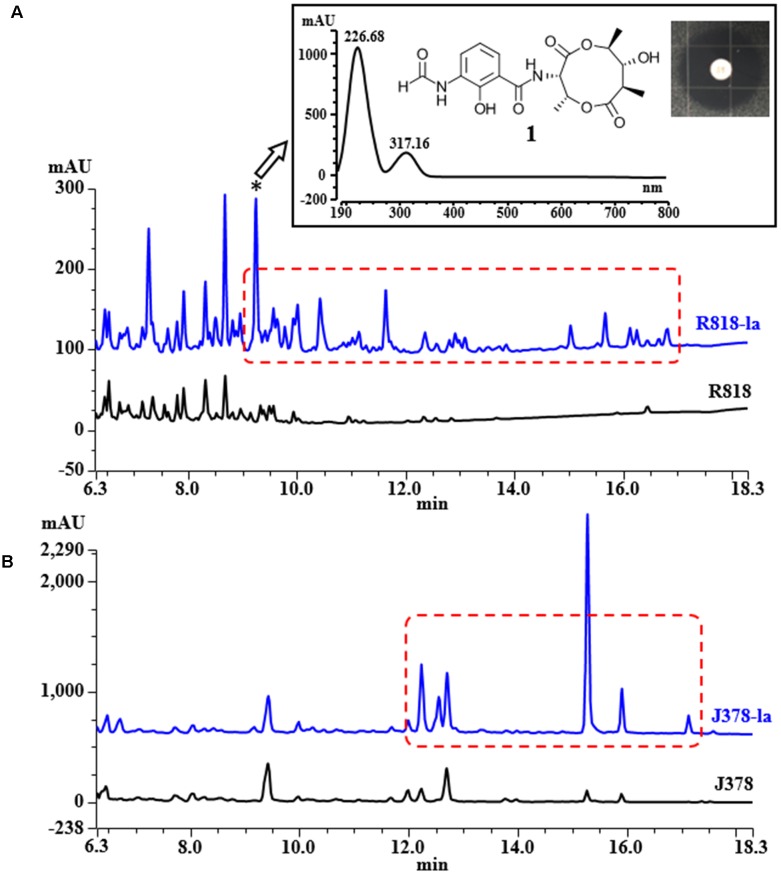
HPLC traces of the extracts from strains R818 **(A)** and J378 **(B)**. Metabolites/peaks elicited or enhanced by the rare earth salt LaCl_3_ (labeled as “-la”) were highlighted by red dashed boxes. Absorbance was recorded at UV = 254 nm. Inset in **A**, the typical UV spectrum of the LaCl_3_-activated metabolites in R818; a representative peak marked by an asterisk (compound **1**, RT = 9.25 min) was purified and identified as urauchimycin D. The inhibition zone of **1** against *C. albicans* was measured as 16 mm.

**FIGURE 3:**
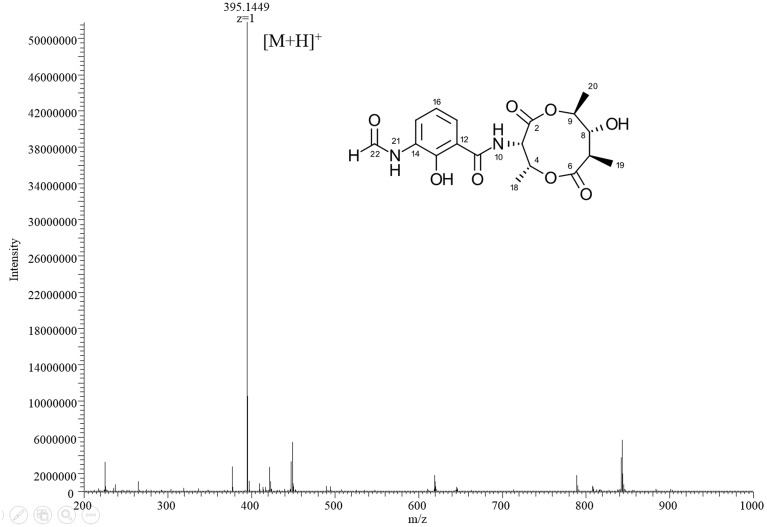
The HR-MS spectrum of compound **1**.

HPLC analysis of these extracts clearly showed new peaks in R818 which might be responsible for the induced antifungal activity in R818. Similarly, peaks with increased abundance in J378 may be associated with the increased activity (**Figure 2**). In the crude extract of R818-la, new peaks with a similar UV spectra (UV_max_ = 227 and 317 nm) were found (**Figure 2A**), which are absent in the crude extract of R818 without the addition of LaCl_3_. In order to identify LaCl_3_-activated metabolites that showed antifungal activity, the strain R818 was fermented in large scale. From 6 L of culture, a representative peak was purified with a retention time (RT) of 9.25 min. This compound (**1**) was isolated as white solid. The *C. albicans* inhibition test showed that compound **1** has an inhibition zone of 16 mm (**Figure 2A**). Using a standard microtiter broth assay method (McCarthy et al., 1992), we observed an MIC of 25 μg/mL for compound **1** against *C. albicans.* Compound **1** also showed moderate anti-*C. difficile* activity (Supplementary Figure S1), whereas the crude extract of R818 showed no activity. HR-MS analysis suggested a molecular formula C_18_H_22_O_8_N_2_ as a [M+H]^+^ ion at *m/z* 395.1449 (**Figure 3**). ^1^H and ^13^C NMR analyses were also performed; the data are summarized in **Table 3** and Supplementary Figures S2, S3. We found the spectroscopic results were identical to those of the known compound urauchimycin D which was also recently isolated from a deep-sea bacterium *Streptomyces somaliensis* SCSIO ZH66 (Yao et al., 2006; Li et al., 2017). Hence, compound **1** was identified as urauchimycin D, one of well-known antifungal antimycins. We concluded that antimycin-type metabolites have been activated by the rare earth salt LaCl_3_ in the strain R818.

**Table 3:** ^1^H and ^13^C NMR chemical shifts of compound **1** in DMSO-*d6*.

Position	δ_H_ (*J* in Hz)	δ_C_
2		169.7
3	5.24 (1H, t, 7.8)	53.8
4	5.50 (1H, m)	70.5
6		175.3
7	2.29 (1H, m)	45.3
8	3.25 (1H, t, 10.8)	77.2
9	4.68 (1H, m)	76.1
10-NH	9.25 (1H, d, 7.8)	
11		169.6
12		114.4
13		150.4
14		126.9
15	8.24 (1H, dd, 1.2, 8.4)	125.1
16	6.93 (1H, t, 8.4)	118.4
17	7.87 (1H, dd, 1.2, 8.4)	123.2
18	1.29 (3H, d, 6.6)	15.1
19	1.17 (3H, d, 6.6)	14.2
20	1.35 (3H, d, 6.6)	18.4
21-NH	9.85 (1H, s)	
22	8.33 (1H, d, 1.8)	160.4
8-OH	5.68 (1H, bs)	

### Activity Toward *C. difficile in Vitro*

One hundred extracts were screened against UK6 in a bacterial growth inhibitory assay at a concentration of 64 μg/mL. UK6 was sensitive to all samples at this concentration. We subsequently performed a broth dilution assay, which showed that 98 of the extracts showed effective bacterial growth inhibition at concentration ≥64 μg/mL, as summarized in **Table 4**. Two samples showed a potent growth inhibitory activity against UK6. Both were extracted from the M864 strain fermented with (WG1-60-60) or without (WG1-60-61) the LaCl_3_ supplementation. The MIC analysis of the M864 extracts indicated that M864 metabolites contain compounds that are more active than the current drugs vancomycin and metronidazole. The MIC value is 0.125 μg/mL for M864 extracts WG1-60-60 and WG1-60-61, whereas it is 0.5 μg/mL for vancomycin and metronidazole (**Table 4**).

**Table 4:** Determination of MIC for the crude extracts, vancomycin, and metronidazole against *C. difficile* UK6 using the broth microdilution method. WG1-60-60 and WG1-60-61 are extracts of M864 fermented with or without the LaCl_3_ supplementation, respectively.

	MIC (μg/mL)									
	0.125	0.25	0.5	1	2	4	8	16	32	64
No. of entries	2	0	2	0	0	0	0	0	0	98
	WG1-60-60		Vancomycin							
	WG1-60-61		Metronidazole							

In order to test cytotoxicity and the therapeutic potential of M864 metabolites, the MTT cell viability assay was performed using HepG2 and HEK cell lines. Inhibition of cell viability by WG1-60-61 was analyzed and the IC_50_ value was determined. As shown in **Table 5**, WG1-60-61 showed low cytotoxicity against HepG2 and HEK293T cell lines with IC_50_ values of 92.37 and 111.6 μg/mL, respectively. The selective index (SI, the rate of IC_50_/MIC) was also calculated. WG1-60-61 showed a good SI against both cell lines, 738.96 for HepG2 cells and 892.8 for HEK293T cells (**Table 5**). These results suggested that M864 metabolites likely contain natural products which are more potent than vancomycin against *C. difficile*, have a good SI, and deserve further investigation.

**Table 5:** Determination of cytotoxicity for the M864 extract WG1-60-61 against HEK293T and hepG2 and cell lines using the MTT cell viability assay.

	MIC (μg/mL)	IC_50_ (μg/mL)		SI (IC_50_/MIC)	
		HEK293T	HepG2	HEK293T	HepG2
WG1-60-61	0.125	111.6	92.37	892.8	738.96

## Conclusion

The less-studied actinobacteria cultivated from deep-sea niches such as those associated with sponges represent unique environment and new taxa for the discovery of novel MNPs. In this preliminary study, we screened extracts of 50 strains, cultivated from diverse marine sponges, mostly collected from the deep sea (∼200 to ∼2,800 fsw). More than half of the strains showed anti-microbial activity in at least one assay, indicating the potential of this group of actinobacteria for the production of natural products. Among them, several strains were identified with exceptional activities, such as R786 and R818 for their potent antifungal activity, J378 for anti-MRSA activity, and N217 for both antifungal and antibacterial activities. Another intriguing strain is M864, the only strain in this work potently inhibiting the growth of *C. difficile*. Recent research has shown the abundance of biosynthesis-like gene clusters in actinobacteria genomes, which do not appear to be expressed under standard laboratory culture conditions. Activation of these cryptic gene clusters would significantly enhance opportunities to discover novel natural products. In this study, a chemical elicitor, LaCl_3_, has been shown effective in inducing antifungal or antibacterial activities in strains that do not show such activities under normal cultivation conditions. In the strain R818, antimycin-type compounds were activated by LaCl_3_, which show potent antifungal activity.

Our results suggest that deep-sea marine actinobacteria represent a promising source of new anti-microbial MNPs. Purification and structural identification of additional bioactive chemicals are in process. The draft genome sequences of R818, J378, and N217 have been determined; analysis of potential biosynthetic gene clusters and gene–compound relationships is also being investigated.

## Author Contributions

DX, LH, CL, QC, DZ, NB, DH, JC, and HZ did the experiments. DX, PM, XS, and GW wrote or edited the manuscript. GW designed the experiments.

## Conflict of Interest Statement

The authors declare that the research was conducted in the absence of any commercial or financial relationships that could be construed as a potential conflict of interest.

## Footnotes

1 https://www.cdc.gov/drugresistance/biggest_threats.html

2 http://www.who.int/mediacentre/news/releases/2017/bacteria-antibiotics-needed/en/

3 https://www.cdc.gov/media/releases/2015/p0225-clostridium-difficile.html
