# Supplementary material for: Bioprospecting Deep-Sea Actinobacteria for Novel Anti-infective Natural Products
Source: Front Microbiol. 2018 Apr 30;9:787. doi: 10.3389/fmicb.2018.00787 (PMC5936781; doi:10.3389/fmicb.2018.00787)
Supplement: Supplementary file 1 [file Data_Sheet_1.pdf]

Supplementary Information

Bioprospecting deep-sea Actinobacteria for novel anti-infective natural products

Dongbo Xu[1, §], Linna Han[1, §], Chunhui Li[2, 3, §], Qi Cao[1], Duolong Zhu[2], Nolan H. Barrett[1], Dedra Harmody[1], Jing Chen[4], Haining Zhu[4], Peter J. McCarthy[1*], Xingmin Sun[2*], Guojun Wang[1*]

[1]Harbor Branch Oceanographic Institute, Florida Atlantic University, Fort Pierce, FL, USA States
[2]Department of Molecular Medicine, Morsani College of Medicine, University of South Florida, Tampa, FL, USA
[3]Infection Control Center, Xiangya Hospital Central South University, Changsha, China
[4]Department of Molecular and Cellular Biochemistry, University of Kentucky, Lexington, KY, USA

[§] These authors contributed to the work equally.

* Correspondence:
Dr. Peter McCarthy, pmccart5@fau.edu
Dr. Xingmin Sun, sun5@health.usf.edu
Dr. Guojun Wang, guojunwang@fau.edu

Methods

The *Clostridium difficile* cell lysis assay was performed as described previously (Ericsson et al., 1960). A single colony UK6 was inoculated into BHIS and cultured at 37°C for 24 h; afterwards, 200 µL of UK6 were mixed with 20 mL of BHIS to make UK6-seeded plates. After solidification, 1.0 µL aliquots of vancomycin or M864 extracts WG1-60-60 and WG1-60-61 with different concentrations, 1 or 2 mg/mL, respectively, were dropped onto the plate. Plates were kept at 37°C in an anaerobic chamber for 24-48 h until the inhibition zone is clearly seen. Methanol is included as a negative control.

References

Ericsson, B.H., Tunevall, G., and Wickman, K. (1960). The paper disc method for determination of bacterial sensitivity to antibiotics: relationship between the diameter of the zone of inhibition and the minimum inhibitory concentration. *Scand. J. Clin. Lab. Invest.* 12, 414-422.

Sfanos, K., Harmody, D., Dang, P., Ledger, A., Pomponi, S., McCarthy, P., and Lopez, J. (2005). A molecular systematic survey of cultured microbial associates of deep-water marine invertebrates. *Syst. Appl. Microbiol.* 28, 242-264. doi: 10.1016/j.syapm.2004.12.002

Results

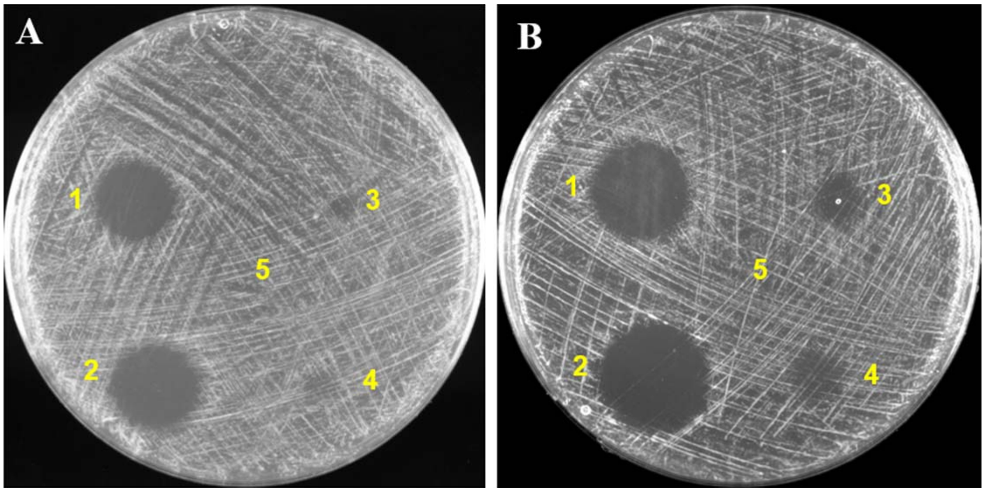

Figure S1. Antibacterial activity of the compound **1** using cell lysis assay. Moderate inhibition of *C. difficile* growth was observed. An aliquot of 2 μL sample solution at either 3.2 mg/mL (**A**) or 6.4 mg/mL (**B**) was dropped onto the UK6-seeded plate. 1, 2: vancomycin; 3, 4: compound **1**; 5: methanol. No inhibition of other bacterial pathogens, *S. aureus*, MRSA and *P. aeruginosa*, are observed.

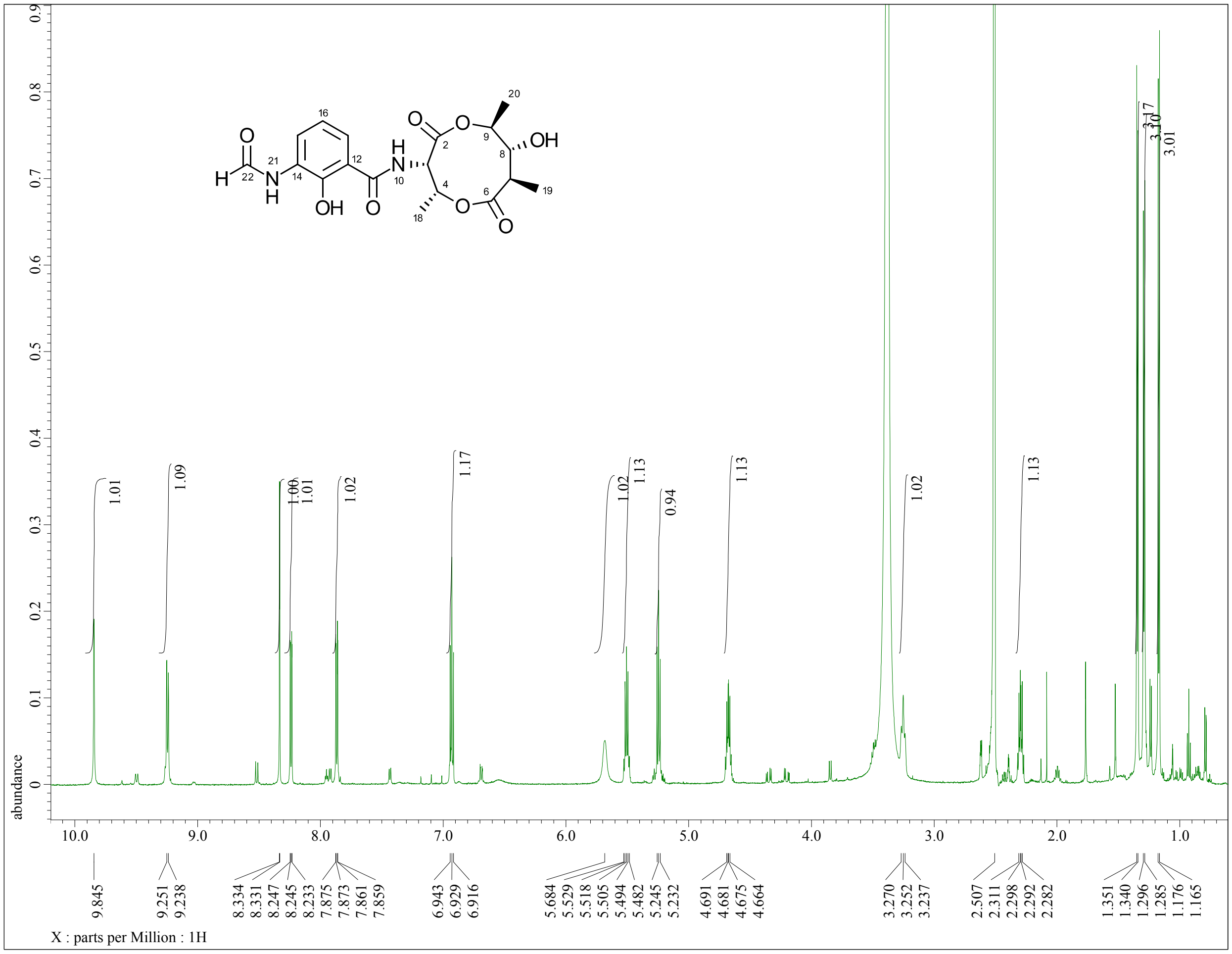

Figure S2. ^{1}H NMR spectrum of compound **1** in DMSO-*d6*.

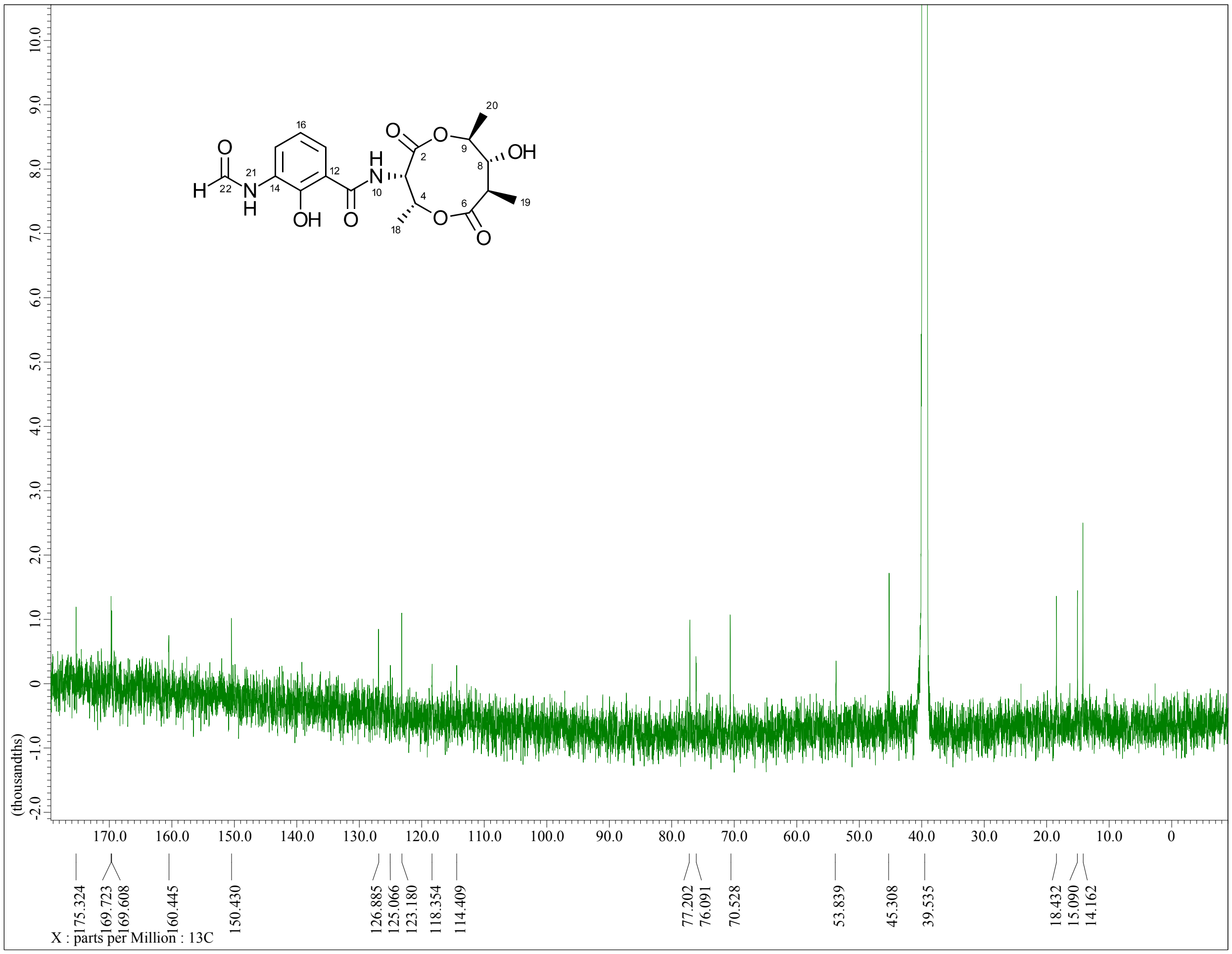

Figure S3. ^{13}C NMR spectrum of compound **1** in DMSO-*d6*.

Table S1. Strains (50) used in this study, taxonomy and their sponge origin.

No.	Strain	Taxonomy *	Accession No.	Sponge origin (Year**)
1	E432	*Pseudonocardia* sp.		*Sarcotagus* sp. or *Smenospongia* sp. (1992)
2	E677	*Streptomyces* sp.		*Ircinia felix* (1992)
3	H882	*Pseudonocardia* sp. SH2		*Thrinacophora funiformis* (1995)
4	J378	*Nocardiopsis* sp.		*Theonella* sp. (1996)
5	J379	*Streptomyces* sp.	AY371434	*Theonella* sp. (1996)
6	J380	*Nocardiopsis alborubida*	AY368532	*Theonella* sp. (1996)
7	J653	*Rhodococcus opacus*		*Spongosorites* sp. (1996)
8	J855	*Gordonia-like* sp.	AY368507	*Spongosorites* sp. (1996)
9	K105	*Pseudonocardia* sp. HUST013		*Discodermia* sp. (1996)
10	K137	*Pseudonocardia* sp. AL041005-10		*Discodermia* sp. (1996)
11	K164	*Pseudonocardia* sp. HUST013		*Discodermia* sp. (1996)
12	K375	*Tsukamurella* sp. FXJ1.531		*Hexactinellida* + *Zoanthidea* (2000)
13	K688	*Rhodococcus ruber* Er I B2.3		*Discodermia* sp. (1997)
14	L128	*Streptomyces* sp. M7		*Discodermia* sp. (1998)
15	L271	*Rhodococcus* sp. CNY-462		*Forcepia* sp. (1999)
16	L733	*Streptomyces* sp.2		*Axinellida* (1999)
17	M864	*Salinispora* sp.		*Oceanapiidae* (1993)
18	N197	*Streptomyces* sp. JVCH105X		*Forcepia* sp. (1999)
19	N201	*Streptomyces* sp.		*Forcepia* sp. (1999)
20	N203	*Streptomyces* sp.		*Forcepia* sp. (1999)
21	N212	*Streptomyces noboritoensis*		*Forcepia* sp. (1999)
22	N217	*Streptomyces* sp. S37		*Forcepia* sp. (1999)
23	N218	*Promicromonospora aerolata*		*Forcepia* sp. (1999)
24	N227	*Promicromonospora endophytica*		*Forcepia* sp. (1999)
25	N248	*Agrococcus casei*		*Forcepia* sp. (1999)
26	N816	*Streptomyces* sp.		*Scleritoderma cyanea* (2000)
27	P114	*Streptomyces* sp. FXJ1.412		*Discodermia* sp. (1996)
28	P183	*Streptomyces* sp. FiRh17		*Discodermia* sp. (1996)
29	P257	*Salinispora arenicola* CNY-685		*Discodermia* sp. (1996)
30	P419	*Streptomyces* sp. FZ92		*Discodermia* sp. (1998)

31	P901	*Pseudonocardia* sp. AL041005-10		*Discodermia* sp. (1998)
32	P947	*Mycobacterium* sp. L1906		*Discodermia* sp. (1998)
33	R529	*Nocardiopsis metallicus*	AY368531	*Gorgonacea* (2000)
34	R622	*Tsukamurella spongiae* K363		*Hexactinellida* + *Zoanthidea* (2000)
35	R783	*Pseudonocardia* sp.		*Forcepia* sp. (2001)
36	R786	*Streptomyces albidoflavus* S19		*Forcepia* sp. (2001)
37	R787	*Streptomyces ambofaciens* OZK18		*Forcepia* sp. (2001)
38	R817	*Streptomyces* sp. S15		*Forcepia* sp. (2001)
39	R818	*Streptomyces* sp.		*Forcepia* sp. (2001)
40	R858	*Pseudonocardia* sp.		*Forcepia* sp. (2001)
41	S181	*Pseudonocardia* sp.		*Discodermia* sp. (2000)
42	S355	*Streptomyces* sp.		*Forcepia* sp. (2001)
43	S579	*Pseudonocardia* sp. EC080619-01		*Forcepia* sp. (2001)
44	S653	*Actinomycetospora* sp.		*Forcepia* sp. (2001)
45	V324	*Streptomyces tendae*		*Theonellidae* n.sp. (2004)
46	V453	*Sphingomanas paucimobilis*		*Leiodermatium* sp. (2005)
47	V459	*Leifsonia* sp.		*Leiodermatium* sp. (2005)
48	V469	*Leifsonia* sp.		*Leiodermatium* sp. (2005)
49	V474	*Leifsonia* sp.		*Leiodermatium* sp. (2005)
50	X306	*Erythrobacter* sp.		*Leiodermatium* sp. (2010)

* Taxonomy is based on Blast analysis of 16S rDNA amplified from each strain.

** The year of sponge sample collection (Sfanos et al., 2005).

Table S2. Disk disffusion assay of extractions against *Candida albicans*; *Staphylococcus aureus* and MRSA, *Pseudomonas aeruginosa*. Inhibition is recorded as the diameter of the zone of growth inhibition (mm).

Strain	Inhibition zone (mm)				Concentration[4]
	C.a.	MRSA	S.a.	P.a.	
E677	9 [1]	11	ND	ND	5
E677-la	10	10	ND	ND	5
J378	ND	12	9	ND	5
J378-la	ND	25	16	ND	5
J855	ND	ND	18	ND	5
J855-la	ND	ND	13	ND	5
L128	12 [1]	10	ND	ND	5
L128-la	ND	9 [1]	ND	ND	5
L733	ND	13	10	ND	5
L733-la	ND	12	10	ND	5
M864	ND	ND	30	ND	5
M864-la	ND	ND	32	ND	5
N197	ND	9 [1]	ND	ND	5
N197-la	ND	10 [1]	ND	ND	5
N200	ND	10	- [2]	ND	10
N200-la	ND	10	- [2]	ND	10
N201	10 [1]	16	- [2]	9	10
N201-la	ND	13	- [2]	ND	10
N203	12	18	- [2]	14	10
N203-la	ND	11	- [2]	ND	10
N217	11	20	14	14	5
N217-la	ND	16	13	10	5
N218	9	10 [1]	- [2]	ND	10
N218-la	8 [1]	10 [1]	- [2]	ND	10
N227	10 [1]	ND	- [2]	ND	10
N227-la	12 [1]	ND	- [2]	ND	10
N248	ND	ND	- [2]	ND	10
N248-la	ND	10	- [2]	ND	10

N816	ND	ND	ND	ND	5
N816-la	ND	11	12	ND [3]	5
P114	ND	9	ND	ND	5
P114-la	13 [1]	10	ND	ND	5
P183	ND	9 [1]	ND	ND	5
P183-la	ND	9 [1]	ND	ND	5
P257	ND	ND	12	ND	5
P257-la	ND	ND	26	ND	5
P419	ND	8	ND	ND	5
P419-la	ND	ND	8	ND	5
P901	9	ND	ND	ND	5
P901-la	ND	ND	ND	ND	5
R786	22	12 [1]	ND	ND	5
R786-la	22	13 [1]	ND	ND	5
R787	ND	8	ND	ND	5
R787-la	ND	8	ND	ND	5
R817	ND	10	ND	ND	5
R817-la	ND	11 [1]	ND	ND	5
R818	ND	ND	- [2]	ND	10
R818-la	21	ND	- [2]	ND	10
S355	ND	ND	- [2]	ND	10
S355-la	ND	10 [1]	- [2]	ND	10
S653	ND	12	- [2]	ND	10
S653-la	ND	9	- [2]	ND	10
V324	ND	11	- [2]	ND	5
V324-la	ND	15	- [2]	ND	5
Positive control	27	19	22	23	

Note: C.a., *C. albicans*; S.a., *S. aureus*; P.a., *P. aeruginosa*. Positive controls are nystatin (100 U) for *C. albicans*; cefoxitin (30 µg) for MRSA; gentamicin (10 µg) for *S. aureus* and *P. aeruginosa*. '-la', cultures supplemented with 2 mM $LaCl_3$; ND, activity not detected; [1], the edge of inhibition zone is hazy; -[2], not determined; [3], anti-P.a. activity was observed only using

partially purified fractions; [4], extractions are dissolved in methanol with a final concentration of 5 or 10 mg/mL.
